# Win and Loss Responses in the Monetary Incentive Delay Task Mediate the Link between Depression and Problem Drinking

**DOI:** 10.3390/brainsci12121689

**Published:** 2022-12-09

**Authors:** Yu Chen, Isha Dhingra, Thang M. Le, Simon Zhornitsky, Sheng Zhang, Chiang-Shan R. Li

**Affiliations:** 1 Department of Psychiatry, Yale University School of Medicine, New Haven, CT 06519, USA; 2 Department of Neuroscience, Yale University School of Medicine, New Haven, CT 06520, USA; 3 Interdepartmental Neuroscience Program, Yale University School of Medicine, New Haven, CT 06520, USA; 4 Wu Tsai Institute, Yale University, New Haven, CT 06520, USA

**Keywords:** alcohol misuse, depression, comorbidity, reward processing, neural markers, monetary incentive delay task, fMRI, AUDIT, BDI-II, mediation analysis

## Abstract

Depression and alcohol misuse, frequently comorbid, are associated with altered reward processing. However, no study has examined whether and how the neural markers of reward processing are shared between depression and alcohol misuse. We studied 43 otherwise-healthy drinking adults in a monetary incentive delay task (MIDT) during fMRI. All participants were evaluated with the Alcohol Use Disorders Identification Test (AUDIT) and Beck’s Depression Inventory (BDI-II) to assess the severity of drinking and depression. We performed whole brain regressions against each AUDIT and BDI-II score to investigate the neural correlates and evaluated the findings at a corrected threshold. We performed mediation analyses to examine the inter-relationships between win/loss responses, alcohol misuse, and depression. AUDIT and BDI-II scores were positively correlated across subjects. Alcohol misuse and depression shared win-related activations in frontoparietal regions and parahippocampal gyri (PHG), and right superior temporal gyri (STG), as well as loss-related activations in the right PHG and STG, and midline cerebellum. These regional activities (*β*’s) completely mediated the correlations between BDI-II and AUDIT scores. The findings suggest shared neural correlates interlinking depression and problem drinking both during win and loss processing and provide evidence for co-morbid etiological processes of depressive and alcohol use disorders.

## 1. Introduction

Depression and alcohol misuse are two leading, comorbid, causes of disability [1,2,3,4,5]. Individuals with depression are more likely to drink to cope with negative mood, elevating the risks in developing an alcohol-use disorder (AUD) [3,6]. Drinkers, especially those with AUDs, often experience depression [7], which in turn leads to more drinking [8,9]. Indeed, co-occurring depression and alcohol misuse are known to dispose individuals to greater severity and more frequent relapse of both conditions [10]. In particular, investigators have suggested a causal pathway whereby alcohol dependence increases the risk of major depression rather than vice versa [1]. Specifically, with alcohol use and depression symptom severity quantified at baseline and 1-year follow-up, the structural equation model with AUD leading to major depression showed the best fit [11]. However, it remains unclear whether this is also true of drinkers with mild to moderate alcohol use severity.

Reward delivers pleasure and drives motivated behaviors. Investigators have employed the monetary incentive delay task (MIDT) or card-guessing task to identify the neural responses to win and loss [12,13]. Individuals with depression relative to controls demonstrated altered reward-related activations [14,15,16]. For instance, blunted striatal activity in response to monetary reward and loss has been reported in patients with depression [17,18,19] and anhedonia [20]. Ventral striatal hypoactivity during anticipation of win and loss was associated with the severity of depression [21]. However, the findings seemed less consistent for the orbitofrontal cortex (OFC), anterior cingulate cortex, and middle frontal gyrus, with greater activation reported for depressed vs. non-depressed individuals in some studies [22,23] but the opposite in others [24,25,26,27,28]. A meta-analysis suggested that individuals with depression may be characterized by less ventral striatum (VS) and higher OFC responses to reward and higher amygdala response to punishment [14]. 

Altered neural activities during reward and punishment processing have also been reported for AUD, although the findings likewise varied. Patients with AUD vs. controls showed reduced VS activation during anticipation of both win and loss in the MIDT [29,30,31] and lower activations in the VS, lateral OFC, medial prefrontal cortex (mPFC), and dorsolateral PFC during win vs. loss in a card-guessing task [32]. In addition, patients with AUD relative to controls showed higher activations in the VS, anterior insula, and mPFC during win outcome and in the insula and lateral frontal cortex during loss outcome in the MIDT [32,33,34], higher VS response to loss outcome in a reward-guessing task [35], and lower superior/middle frontal cortical responses to loss outcome in a risk-taking task [36]. Other studies showed higher activations in the VS, anterior cingulate cortex, and paracentral and postcentral gyri during anticipation of reward in patients with AUD as compared to controls [37] and no differences in activation to reward outcome between patients with AUD and controls [32,37], or between those with and without a family history of alcoholism [38]. 

In summary, many studies have reported altered regional brain activities during reward/punishment processing in both depression and AUD. However, the findings varied substantially across studies, even for the VS, a hub of the reward circuit. Differences in MIDT paradigms [39] and clinical heterogeneity may account for the discrepancy in findings. Further, it is far from clear, based on these findings, whether alcohol misuse and depression shared neural correlates during reward and punishment processing. Thus, it would seem important to evaluate both depression and drinking severity in the same cohort of individuals.

In the current study, we aimed to investigate the neural correlates of reward and punishment processing shared between problem drinking and depression in a sample of largely nondependent alcohol drinkers. We performed whole-brain regressions to examine regional responses to win and loss outcomes in an MIDT each in association with the severity of problem drinking and with depression. Further, we employed mediation analyses to evaluate the inter-relationships between the shared neural correlates, drinking, and depression severity. We hypothesized that the shared neural correlates would mediate the relationships between problem drinking and depression. 

## 2. Methods

### 2.1. Subjects

Forty-three adult alcohol drinkers (sixteen women; 23–74 or 45.4 ± 12.9 years of age) participated in this study. All subjects were otherwise healthy with no current medical conditions or use of prescription medications. None reported a history of head injury or neurological illness. Other exclusion criteria included current or past Axis I Disorders, including dependence on a psychoactive substance (except alcohol), according to DSM-IV [40]. The Human Investigation Committee at the Yale University School of Medicine approved the study (protocol code 0906005272). All subjects gave written informed consent prior to participation.

### 2.2. Assessments, Monetary Incentive Delay Task (MIDT), and Imaging Protocol

**Figure 1:**
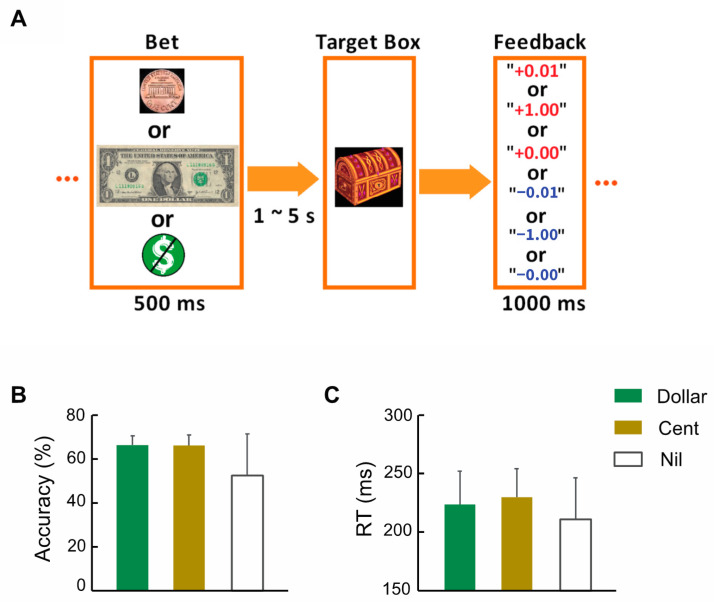
(**A**) Monetary incentive delay task: Each trial starts with a bet (a dollar, a cent, or no money). After a randomized interval of 1–5 s, a target box is presented and disappears after a response window. Subjects are requested to make a response as quickly as possible to collect the money (win) before it disappears, following by feedback that indicates the amount of money won (in red) or lost (in blue). (**B**) Accuracy rate and (**C**) RT of trials (mean ± SD).

All participants were evaluated with the Alcohol Use Disorders Identification Test (AUDIT), Beck’s Depression Inventory (BDI-II), and the Fagerström Test for Nicotine Dependence (FTND). A 10-item instrument to screen for harmful drinking, the AUDIT, assesses the frequency of alcohol consumption, dependence, and associated harm. Each item is scored from 0 to 4 and the total score ranges from 0 to 40, with a higher score indicating greater severity of problematic alcohol use [41,42]. Five of the forty-three participants with the highest AUDIT scores (all > 14) also met criteria for alcohol abuse. The BDI-II is a 21-item assessment of the presence and severity of depression symptoms within the prior 2 weeks, with each item scored 0 to 3. A total score of 0 to 13, 14 to 19, 20 to 28, and 29 to 63 indicates minimal, mild, moderate, and severe depression, respectively [43,44,45]. Three of the forty-three participants scored > 19 and had moderate severity of depression. The FTND assesses the severity of cigarette consumption, compulsion to smoke, and physical dependence on nicotine, with a range of 0–10. A higher FTND score indicates greater severity of nicotine dependence [46]. Each subject completed two 10-min runs of the MIDT (Figure 1A), as described in our previous studies [47,48]. Across subjects, they completed an average of 184 ± 4 (mean ± SD) trials.

### 2.3. Imaging Data Preprocessing and Group Analyses

Briefly, brain images were collected using multiband imaging (multiband factor = 3) with a three-Tesla MR scanner (Siemens Trio, Erlangen, Germany). Data were analyzed with Statistical Parametric Mapping (SPM8, Wellcome Department of Imaging Neuroscience, University College London, UK), including realignment, slice timing, co-registration, segmentation, normalization, and smoothing, as in our earlier studies [39,48]. We examined event-related BOLD signals in a single model focusing on the feedback or outcome phase of win or loss processing, as described in our previous study [47]. We performed one-sample *t* tests of win vs. nil and loss vs. nil. To investigate the neural correlates of AUDIT and BDI-II, we conducted whole-brain linear regressions of these contrasts on AUDIT and BDI-II, separately, with age, sex, and FTND scores as covariates. All models were evaluated with a threshold combining voxel *p* < 0.001, uncorrected, and cluster *p* < 0.05 family-wise error (FWE), corrected, following current reporting standards. Voxels with peak activity were indicated with Montreal Neurological Institute (MNI) coordinates. We performed inclusive masking to identify the neural correlates shared between AUDIT and BDI-II for win vs. nil and loss vs. nil, respectively.

### 2.4. Mediation Analyses

We examined how activations of the regions of interest, AUDIT and BDI-II scores were inter-related with mediation analyses [49], as described in our prior work [50]. The mediation test was performed by employing three regression equations [49].

$$Y=i_{1}+cX+e_{1}$$

$$Y=i_{2}+c^{\prime }X+bM+e_{2}$$

$$M=i_{3}+aX+e_{3}$$

where *a*, *b*, *c*′, and *c* represent path coefficients, and variable M is a mediator of the correlation X → Y. The significant paths *a* and *b*, as well as (*c*—*c*′), indicate that X → Y is mediated by M. Moreover, if the path *c*′ is not significant, then X → Y is completely mediated by M.

## 3. Results

### 3.1. Clinical Characteristics and Behavioral Performance

**Figure 2:**
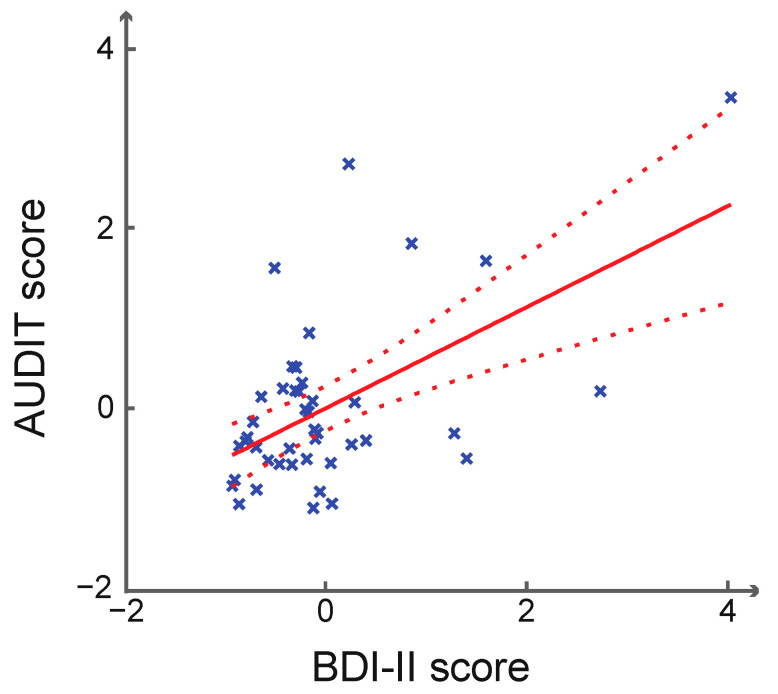
Scatter plot of AUDIT and BDI-II scores. Note that the standardized residual scores after controlling for age, sex, and FTND score as covariates are shown here. Dashed lines represent 95% confidence intervals of the mean regression (solid line). Crosses represent individual data points.

Table 1 summarizes the demographic and clinical characteristics for men and women separately. The mean FTND score was <1, suggesting a largely non- or light-smoking sample. No sex differences were noted for age, years of education, AUDIT, BDI-II, or FTND score; thus, we combined men and women in data analyses. Figure 1B,C show the accuracy rate and reaction time (RT) of dollar, cent, and nil trials. The accuracy rates were close to 67%, suggesting the success of the staircase procedure. Across subjects, the AUDIT score was significantly and positively correlated with the BDI-II score without (*r* = 0.555, *p* < 0.001) or with (*r* = 0.560, *p* < 0.001; Figure 2) age, sex, and FTND as covariates. The AUDIT or BDI-II score did not show a significant correlation with either accuracy rate or with RT of dollar, cent, and nil trials (*p’s* ≥ 0.062 without covariates; *p’s* ≥ 0.109 with age, sex, and FTND as covariates), or the differences of dollar/cent vs. nil in either accuracy rate or RT (*p’s* ≥ 0.338 without covariates; *p’s* ≥ 0.308 with age, sex and FTND as covariates). 

### 3.2. Brain Activations of Win vs. Nil and Loss vs. Nil

In one-sample *t*-tests, we evaluated regional activations to win vs. nil and loss vs. nil across all subjects. The results are shown in Supplementary Figure S1 and summarized in Supplementary Table S1. Compared to nil, win trials showed higher activations in the bilateral caudate, anterior cingulate cortex, bilateral lingual gyri, and cerebellum; loss trials showed higher activations in the bilateral lingual gyri, anterior cingulate cortex, bilateral insula, bilateral precentral gyri, and cerebellum. 

### 3.3. Whole Brain Regressions on AUDIT and BDI-II Scores

**Figure 3:**
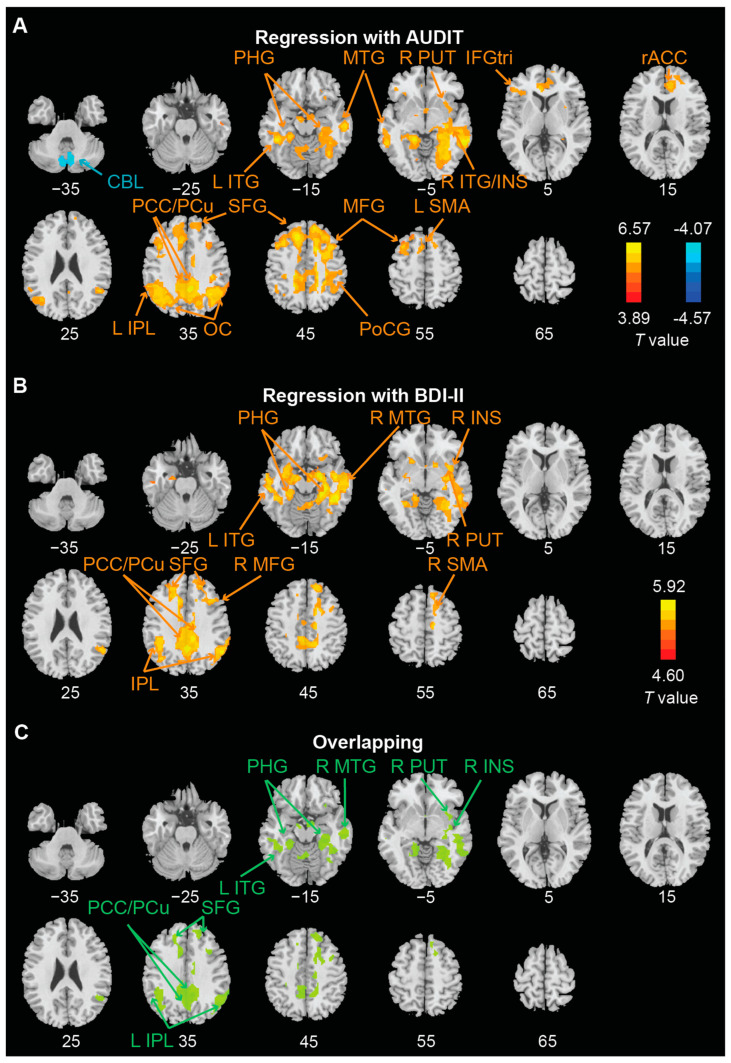
Regional activations to win vs. nil in correlation with (**A**) AUDIT score and (**B**) BDI-II score with age, sex, and FTND score as covariates with threshold of voxel *p* < 0.001 in combination with cluster-level *p* < 0.05 FWE corrected; The brain regions are summarized in Table 2. (**C**) ROIs overlapped of (**A**,**B**). Warm/cool colors indicate positive/negative correlations. L: left; R: right; CBL: cerebellum; IFGtri: inferior frontal gyrus pars triangularis; INS: insula; IPL: inferior parietal lobule; ITG: inferior temporal gyrus; MFG: middle frontal gyrus; MTG: middle temporal gyrus; OC: occipital cortex; Pcu: precuneus; PHG: parahippocampal gyrus; PoCG: postcentral gyrus; PUT: putamen; rACC: rostral anterior cingulate cortex; SFG: superior frontal gyrus; SMA: supplementary motor area.

**Figure 4:**
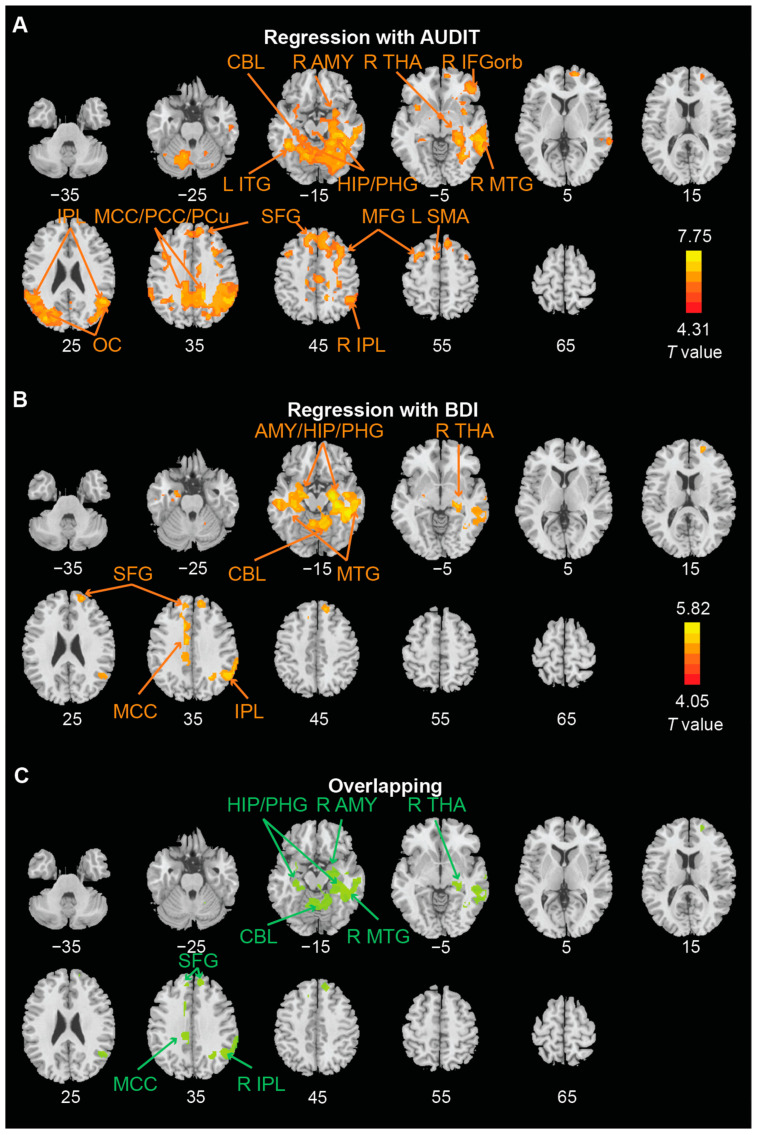
Regional activations to loss vs. nil in correlation with (**A**) AUDIT and (**B**) BDI-II score with age, sex, and FTND score as covariates at voxel *p* < 0.001 in combination with cluster-level *p* < 0.05 FWE corrected. The brain regions are summarized in Table 2. (**C**) ROIs overlapped of (**A**,**B**). L: left; R: right; AMY: amygdala; CBL: cerebellum; IFGorb: inferior frontal gyrus pars orbitalis; HIP: hippocampus; IPL: inferior parietal lobule; ITG: inferior temporal gyrus; MCC: mid-cingulate cortex; MFG: middle frontal gyrus; MTG: middle temporal gyrus; OC: occipital cortex; PHG: parahippocampal gyrus; SFG: superior frontal gyrus; SMA: supplementary motor area; THA: thalamus.

We performed whole-brain regressions of win > nil (Figure 3A,B; Table 2) and loss > nil (Figure 4A,B; Table 2) against AUDIT and BDI-II scores, separately, with age, sex, and FTND score as covariates across subjects. We identified the clusters that overlapped between AUDIT and BDI-II regressions (Figure 3C and Figure 4C). For win > nil, the overlapping clusters included bilateral parahippocampal gyrus, superior frontal gyrus, and posterior cingulate cortex/precuneus, left inferior parietal lobule and inferior temporal gyrus, and right middle temporal gyrus, putamen, and insula. For loss > nil, the overlapping clusters included bilateral hippocampus/parahippocampal gyrus, superior frontal gyrus, mid-cingulate cortex, cerebellum, and right amygdala, middle temporal gyrus, thalamus, and inferior parietal lobule.

### 3.4. Mediation Models

**Figure 5:**
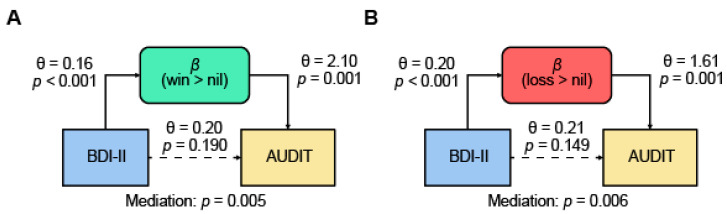
Mediation models: brain activation (*β*) of (**A**) win > nil (in green) and (**B**) loss > nil (in red) of the shared ROIs completely mediated the correlation between BDI-II (in blue) and AUDIT (in yellow) scores. All six models were assessed for each contrast and evaluated at a corrected threshold *p* = 0.05/6 = 0.0083. The *p* values associated with mediation are for the path “a × b” (see Section 2). The statistics of all models are summarized in Table 3.

The AUDIT and BDI-II scores were positively correlated, as shown earlier. As expected, the activation (*β*) of overlapping clusters was each positively correlated with the AUDIT score (*r* = 0.66, *p* < 0.001 for win > nil and *r* = 0.65, *p* < 0.001 for loss > nil) and BDI-II score (*r* = 0.67, *p* < 0.001 for win > nil and *r* = 0.64, *p* < 0.001 for loss > nil). Thus, we conducted mediation analyses to examine the relationships amongst the βs, AUDIT, and BDI-II scores. We tested all six models for each contrast. The results showed that the *β* of win > nil and of loss > nil each completely mediated the relationship between BDI-II and AUDIT (Figure 5). None of the other models showed significant mediation at a corrected threshold *p* < 0.05/6 = 0.0083 (Table 3). 

## 4. Discussion

We identified regional brain responses to monetary win and loss outcomes in correlation with both AUDIT and BDI-II scores in non-dependent drinkers. Individuals with higher AUDIT and BDI-II scores showed greater activation to wins in bilateral fronto-parietal cortex, precuneus/posterior cingulate cortex, and right temporal cortex. Individuals with higher AUDIT and BDI scores also showed higher activation to losses in bilateral (but predominantly right-hemispheric) hippocampus/parahippocampal gyrus, cerebellum, right temporal and inferior parietal cortex, and posterior cingulate cortex. These regional activities completely mediated the relationship between depression and alcohol use severity. The findings highlight shared neural correlates of reward and punishment processing between depression and alcohol misuse and may help research of the etiologies of comorbid depression and AUD. We highlight the major findings for discussion. 

### 4.1. Depression and Alcohol Misuse Shared Neural Responses to Monetary Win and Loss

A wide array of cortical and subcortical regions was involved during win and loss processing in link with depression and problem drinking. Most notable among these regions are bilateral hippocampi/parahippocampal gyri (HC/PHG), which are shared for both contrasts—win vs. nil and loss vs. nil—although the latter also involved the right amygdala in activities shared between depression and alcohol misuse. The HC/PHG is best known for its function in memory encoding and retrieval, and high-arousing, salient events consistently engage the HC/PHG [51,52]. Relative to nil, both win and loss trials are more salient; thus, the current findings suggest elevated HC/PHG responses to saliency both in association with the severity of depression and alcohol misuse. These findings are broadly consistent with previous reports of HC/PHG structural and functional changes in depressive and anxiety [53,54,55] and alcohol use [56,57,58] disorders. Studies of the etiological mechanisms of depression have emphasized ill-adaptive HC/PHG circuit responses to stress [59]. An imaging literature has associated with HC/PHG circuit dysfunction in emotional and reward processing [60,61,62]. Here, we demonstrated that depression and alcohol misuse both implicate the HC/PHG in heightened responses to wins and losses—salient stimuli irrespective of their valence.

The shared correlates that appeared to be specific to win and loss processing are the posterior cingulate cortex and precuneus (PCC/Pcu) and mid-cingulate cortex (MCC), respectively. Although less of a focus in human studies of reward processing, the PCC is implicated in post-decisional reward signaling in neuronal recordings from behaving monkeys [63]. Notably, the PCC/Pcu did not show significantly higher responses to win vs. nil in the one-sample *t* test (Supplementary Figure S1), suggesting this default mode network region solely as a correlate of individual variation in depression and alcohol misuse. In contrast, a hub of the limbic motor circuit, the MCC responds to learning of aversive consequences and behavioral avoidance [64,65]. The MCC along with other midline brain regions, including the supplementary motor area, showed significantly higher activation to loss vs. nil in the one-sample *t* test (Supplementary Figure S1). Thus, individuals with more severe depression and alcohol misuse would engage the MCC greater than the average extent, likely to support emotional motor processes of negative reinforcement. Also notable is the rostral anterior cingulate cortex (rACC), which showed higher responses to both win and loss vs. nil across subjects but only higher responses to win vs. nil in correlation with AUDIT but not BDI-II score. The rACC is part of the saliency and executive control circuit [66,67]; rACC responses to reward may potentially represent a unique marker of alcohol misuse. These findings support studies of neuromodulation of the ACC as a treatment of AUD [68,69]. More research is warranted to evaluate sub-regional cingulate cortical responses to reward and punishment and how these responses may be altered in depressive and alcohol use disorders [70]. 

### 4.2. Shared Neural Responses Mediated the Link of Depression and Alcohol Misuse

Across individuals, BDI-II and AUDIT scores were positively correlated, supporting the comorbidity of depression and problem drinking even in non-dependent drinkers [7,10,71,72,73,74]. Importantly, we observed shared brain activities in response to reward and to punishment, and these activities completely mediated the relationships between the severity of depression and alcohol misuse. Specifically, with correction for multiple testing, the findings suggest that depression contributes to alcohol misuse through the shared neural responses to monetary win and loss. As discussed in the Introduction, earlier studies have suggested individual differences in reward sensitivity as a risk factor of both depression and alcohol misuse [75,76,77]. The current findings expand this literature by showing evidence that depression contributes to alcohol drinking through brain activities in response to reward and punishment. Notably, although only one of the mediation models—depression → *β* of win or loss → problem drinking—showed significant, complete mediation, the model problem drinking → *β* of win or loss → depression showed an incomplete mediation effect with a *p* < 0.009. Given the small sample size of the study, it remains to be seen whether the shared correlates may mediate the link of depression and problem alcohol use bidirectionally. 

### 4.3. Limitations of the Study and Future Directions

Several limitations need to be noted for the study. Firstly, the study comprised a small sample and, though evaluated at a corrected threshold, the findings and particularly those of regional responses shared between AUDIT and BDI-II regressions, need to be replicated. For the same reason, we did not investigate potential sex differences in the current findings or whether depression and alcohol misuse may be associated with shared reduction in neural responses differentiating reward and punishment [78]. Secondly, reward and punishment come in many forms. Thus, the findings should be considered specific to monetary win and loss. It remains unclear whether or how neural processes of other modalities of reward and punishment (e.g., social interaction and rejection) may be shared in the etiological processes of depression and alcohol misuse. Lastly, although the participants in the current study were largely non- or light smokers (mean FTND score < 1), we cannot entirely rule out the effects of smoking on the current findings. Studies of a larger sample and perhaps of individuals with varying levels of nicotine dependence would be needed to address the effects.

Although the study focused on reward processing, depression and alcohol misuse involve and may share other etiological processes, including fronto-limbic dysfunction in impaired inhibitory control and emotion processing and learning [79,80,81,82,83] and altered mitochondrial bioenergetics [84,85,86]. More studies are needed to evaluate the respective and potentially interactive roles of these neural and biological mechanisms of depression and alcohol misuse. Further, to the extent that these findings are considered specifically for non-dependent drinkers, future studies may incorporate assessment of physical, social, and cultural factors that may influence alcohol intake and the emotional context under which individuals engage in use and misuse of alcohol and “alcohol-like” drinks [87,88].

## 5. Conclusions

To conclude, we showed a significant correlation between the severity of depression and alcohol misuse in non-dependent drinkers. Neural responses to monetary wins and losses both mediated the relationship between depression and problem drinking. Suggesting shared etiological processes, these findings not only enhance our understanding of the neural mechanisms associated with both psychiatric conditions but also provide evidence for the importance of concurrent treatment of depression and alcohol misuse in clinical populations. 

## Figures and Tables

**Table 1:** Demographic and clinical characteristics in men and women.

	Men (*n* = 27)	Women (*n* = 16)	*t*	*p*
Age (years)	44.9 ± 11.3	46.4 ± 15.6	−0.34	0.71
Education (years)	15.0 ± 3.6	14.8 ± 3	0.27	0.79
AUDIT score	7.8 ± 9.3	4.9 ± 4.8	1.15	0.26
BDI-II score	5.0 ± 8.7	7.7 ± 8.8	−0.96	0.34
FTND score	0.4 ± 1.4	0.8 ± 2.2	−0.63	0.53

Note: Values of mean ± SD; AUDIT: Alcohol Use Disorders Identification Test; BDI-II: Beck’s Depression Inventory; FTND: Fagerström Test for Nicotine Dependence.

**Table 2:** Regional activations to win > nil and loss > nil in correlation with AUDIT and BDI-II scores with age, sex, and FTND score as covariates.

Volume (mm^3^)	Peak Z	MNI Coordinate (mm)			Side	Identified Brain Region
		x	y	z		
Regression with AUDIT						
Positive with Win > Nil						
100,845	5.34	−15	29	43	L/R	DLPFC, SFG, Precentral G, SMA
	4.84	36	−31	40	L/R	IPL, PCC, Precuneus
49,815	5.27	−21	−37	−11	L	Hippocampus, Amygdala
	5.21	54	−46	−5	R	Temporal G
6669	4.48	6	41	13	L/R	ACC
2862	4.06	−9	−7	−11	L/R	Hypothalamus
2511	3.85	−33	32	4	L	IFG
Negative with Win > Nil						
4266	4.06	6	−70	−35	L/R	CBL
Positive with Loss > Nil						
68,796	5.97	54	−31	−8	R	Temporal G, Hippocampus, Amygdala, Hypothalamus
	5.29	−12	−58	−20	L	CBL
84,375	5.10	18	−46	40	L/R	Precuneus, DLPFC, MPFC, MCC
	4.90	60	−55	31	R	IPL, Precentral G,
3078	4.49	−33	11	−8	L	Insula
4806	4.30	−33	−1	40	L	Precentral gyrus
2538	4.30	9	59	−5	R	VMPFC
Negative with Loss > Nil						
None						
Regression with BDI						
Positive with Win > Nil						
54,324	4.95	21	−7	−17	R	Hippocampus, Amygdala, Hypothalamus
	4.76	45	−43	−14	R	Temporal G
33,696	4.63	−6	−40	40	L	Precuneus, PCC, DLPFC, SFG, SMA
6048	4.57	57	−61	34	R	IPL
4401	4.29	−42	−40	34	L	IPL
6399	4.26	−12	23	40	L	SFG
Negative with Win > Nil						
None						
Positive with Loss > Nil						
10,665	4.89	−18	−10	−20	L	Hippocampus, Amygdala, Temporal G
25,326	4.81	24	−10	−14	R	Hippocampus, Amygdala, Temporal G, CBL
6399	4.21	63	−46	37	R	IPL
4320	4.08	21	59	22	R	SFG
4158	3.84	−9	−7	34	L	MCC
2403	3.67	−6	−37	40	L	MCC
Negative with Loss > Nil						
None						

Note: Results were evaluated at voxel *p* < 0.001 and cluster-level *p* < 0.05, FWE corrected; L: left; R: right; G: gyrus; Cerebellum: CBL; DLPFC: dorsolateral prefrontal cortex; SFG: superior frontal gyrus, IPL: inferior parietal lobule; PCC: posterior cingulate cortex; ACC: anterior cingulate cortex; IFG: inferior frontal gyrus; MPFC: medial prefrontal cortex; MCC: mid-cingulate cortex; VMPFC: ventromedial prefrontal cortex; SMA: supplementary motor area.

**Table 3:** Mediation models of AUDIT, BDI-II score, and brain activation (*β*) each for win > nil and loss > nil.

			*p* Values				
X	M	Y	X → M	M → Y	X → Y	Mediated X → Y	Mediation
Win > Nil							
AUDIT	BDI	Brain	<0.001	0.006	<0.001	<0.001	0.021
Brain	BDI	AUDIT	<0.001	0.190	<0.001	<0.001	0.204
AUDIT	Brain	BDI	<0.001	0.006	<0.001	0.190	0.013
BDI	Brain	AUDIT	<0.001	<0.001	<0.001	0.190	0.005 *
Brain	AUDIT	BDI	<0.001	0.190	<0.001	0.006	0.201
BDI	AUDIT	Brain	<0.001	<0.001	<0.001	0.006	0.009
Loss > Nil							
AUDIT	BDI	Brain	<0.001	0.010	<0.001	<0.001	0.029
Brain	BDI	AUDIT	<0.001	0.149	<0.001	<0.001	0.166
AUDIT	Brain	BDI	<0.001	0.010	<0.001	0.149	0.020
BDI	Brain	AUDIT	<0.001	<0.001	<0.001	0.149	0.006 *
Brain	AUDIT	BDI	<0.001	0.149	<0.001	0.010	0.162
BDI	AUDIT	Brain	<0.001	<0.001	<0.001	0.010	0.009

Note: The mediation models were evaluated at a corrected *p* = 0.05/6 = 0.0083. * *p* < 0.0083. A significant mediation and non-significant mediated X **→** Y suggest that M completely mediates the correlation of X and Y.

## Data Availability

The data and codes will be shared on request to the corresponding author.

## Supplementary Materials

The following supporting information can be downloaded at: https://www.mdpi.com/article/10.3390/brainsci12121689/s1, Figure S1: Regional activations to (A) Win vs. Nil and (B) Loss vs. Nil. Color bars indicate voxel T values. Results were evaluated at voxel *p* < 0.001 in combination with cluster-level *p* < 0.05 family-wise error (FWE) corrected. The clusters are summarized in Supplementary Table S1. L: left; R: right; ACC: anterior cingulate cortex; AMY: amygdala; CB: caudate body; CBL: cerebellum; dACC: dorsal ACC; IFGorb: inferior frontal gyrus (pars orbitalis); INS: insula; IPG: inferior parietal gyrus; MCC: mid-cingulate cortex; mOFC: medial orbitofrontal cortex; OC: occipital cortex; PCC: posterior cingulate cortex; PoCG: postcentral gyrus; SMG: supramarginal gyrus; SPG: superior parietal gyrus; STR: striatum; TAL: thalamus; Table S1: Regional activations to Win vs. Nil and Loss vs. Nil in the MIDT (n = 43).
