# Supplementary material for: Win and Loss Responses in the Monetary Incentive Delay Task Mediate the Link between Depression and Problem Drinking
Source: Brain Sci. 2022 Dec 9;12(12):1689. doi: 10.3390/brainsci12121689 (PMC9775947; doi:10.3390/brainsci12121689)
Supplement: Supplementary file 1 [file brainsci-12-01689-s001.zip › brainsci-2082478-supplementary.pdf]

SUPPLEMENT

Chen et al., Win and loss responses in the monetary incentive delay task mediate the link between depression and problem drinking

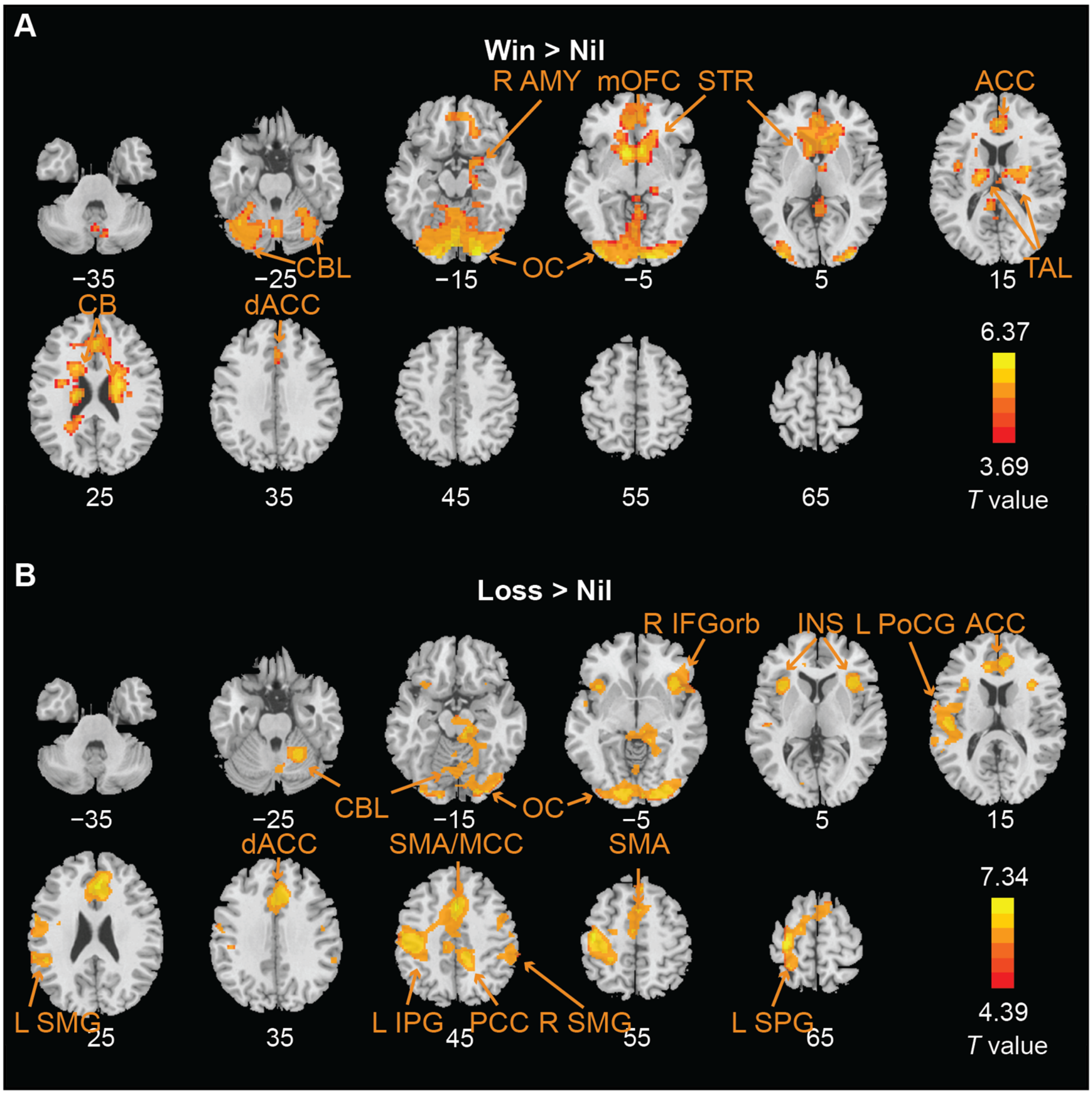

Supplementary Figure S1. Regional activations to **(A)** Win vs. Nil and **(B)** Loss vs. Nil. Color bars indicate voxel *T* values. Results were evaluated at voxel $p < 0.001$ in combination with cluster-level $p < 0.05$ family-wise error (FWE) corrected. The clusters are summarized in Supp. Table S1. L: left; R: right; ACC: anterior cingulate cortex; AMY: amygdala; CB: caudate body; CBL: cerebellum; dACC: dorsal ACC; IFGorb: inferior frontal gyrus (pars orbitalis); INS: insula; IPG: inferior parietal gyrus; MCC: mid-cingulate cortex; mOFC: medial orbitofrontal cortex; OC: occipital cortex; PCC: posterior

cingulate cortex; PoCG: postcentral gyrus; SMG: supramarginal gyrus; SPG: superior parietal gyrus; STR: striatum; TAL: thalamus.

Supplementary Table S1: Regional activations to Win vs. Nil and Loss vs. Nil in the MIDT (n=43).

**volume**	**peak voxel**	**MNI coordinate (mm)**			**side**	**identified brain region**
**(mm^3)**	**(Z)**	**x**	**y**	**z**		
Win > Nil						
34,776	5.85	-9	11	-5	L	Caudate, anterior cingulate cortex
	5.54	6	11	-5	R	Caudate, anterior cingulate cortex
71,577	5.70	21	-88	-14	R	Lingual gyrus
	5.59	-15	-94	-8	L	Lingual gyrus
	5.58	-24	-82	-20	L	Cerebellum
Nil > Win						
None						
Loss > Nil						
33,831	5.30	21	-91	-8	R	Lingual gyrus
	5.08	-18	-94	-11	L	Lingual gyrus
	4.65	21	-52	-23	R	Cerebellum
75,141	5.28	-33	-25	67	L	PrCG/PoCG/IPG/MCC/SMA
	5.10	9	41	22	L/R	Anterior cingulate cortex
5,427	5.01	-33	20	13	L	Insula
8,424	4.75	33	20	13	R	Insula
5,373	3.91	45	-7	40	R	Supramarginal gyrus
Nil > Loss						
None						

Note: voxel $p < 0.001$ and cluster-level $p < 0.05$, FWE corrected; R: right; L: left.
